# The hidden cost of not having a dual diagnosis team (an audit looking at inpatient admissions for Redbridge Community Recovery Team West)

**DOI:** 10.1192/bjo.2021.217

**Published:** 2021-06-18

**Authors:** Madeeha Bandukda, Muhammad Aadil Bhenick, Najam Chaudry, Henok Getachew

**Affiliations:** NELFT

## Abstract

**Aims:**

Co-existing mental illness and substance misuse is highly prevalent within the UK, with approximately 40% of people diagnosed with psychosis having a history of substance misuse. However, in Redbridge we currently do not have access to a dual diagnosis team or integrated care.

This audit aims to assess the health and social implications of fragmented care, plus the effectiveness of mental health services in assessing patients with dual diagnosis and referring to specialist misuse teams. We used the NICE guidelines on co-existing severe mental illness and substance misuse [CG120] to help guide our recommendations.

**Method:**

We identified 50 out of 359 patients within our service who were admitted to psychiatric hospital over a one year period (between 01/11/2019- 01/11/2020).

We looked at medication compliance, use of the Mental Health Act and accommodation status to compare between those with and without known dual diagnosis. We used frequency and length of admission as indicators of how successfully patients were being managed in the community and the cost to the hospital trust. Urine drug screening and referral to substance misuse services were chosen as markers of whether patients were being appropriately managed on admission.

**Result:**

A higher percentage of patients with dual diagnosis were detained under the Mental Health Act compared to those without substance misuse (89% versus 72%). They were more likely to have no fixed abode (28% versus 13%) and be non-compliant with treatment pre-admission (83% versus 56%). Patients with dual diagnosis also had a higher number of hospital admissions, with a greater proportion having 3 admissions that year (11% versus 3%).

Only 50% of patients with known dual diagnosis had a urine drug screen performed on admission and just 25% of patients who were currently misusing substances were referred to specialist services by the inpatient team.

**Conclusion:**

Our audit found that there are overall poorer outcomes for patients with dual diagnosis versus a psychiatric illness only. It is evident that integration of services will improve the care we are able to provide and reduce costs associated with multiple admissions to hospital.

We identified three key areas for improvement. Firstly, we advised on the need to improve documentation. Additionally, we recommend ensuring assessment of current drug misuse is done on admission, including performing simple tests such as urine drug screening. Finally, we highlighted the need to improve discussions about substance misuse with patients, within teams and between services, aiming for integrated and holistic care.

